# Docosahexaenoic Acid Inhibits *Helicobacter pylori* Growth *In Vitro* and Mice Gastric Mucosa Colonization

**DOI:** 10.1371/journal.pone.0035072

**Published:** 2012-04-17

**Authors:** Marta Correia, Valérie Michel, António A. Matos, Patrícia Carvalho, Maria J. Oliveira, Rui M. Ferreira, Marie-Agnès Dillies, Michel Huerre, Raquel Seruca, Ceu Figueiredo, Jose C. Machado, Eliette Touati

**Affiliations:** 1 Institute of Molecular Pathology and Immunology, University of Porto, Porto, Portugal; 2 Faculty of Medicine, University of Porto, Porto, Portugal; 3 Unité de Pathogenèse de Helicobacter, Institut Pasteur, Paris, France; 4 Serviço de Microscopia Electrónica, Hospital Curry Cabral, Lisboa, Portugal; 5 Plate-forme Transcriptome et Epigénome, Génopole Institut Pasteur, Paris, France; 6 Unité de Recherche et d'Expertise en Histotechnologie et Pathologie, Institut Pasteur, Paris, France; 7 Centre for Environmental and Marine Studies (CESAM), Aveiro University, Aveiro, Portugal; Monash University, Australia

## Abstract

*H. pylori* drug-resistant strains and non-compliance to therapy are the major causes of *H. pylori* eradication failure. For some bacterial species it has been demonstrated that fatty acids have a growth inhibitory effect. Our main aim was to assess the ability of docosahexaenoic acid (DHA) to inhibit *H. pylori* growth both *in vitro* and in a mouse model. The effectiveness of standard therapy (ST) in combination with DHA on *H. pylori* eradication and recurrence prevention success was also investigated. The effects of DHA on *H. pylori* growth were analyzed in an *in vitro* dose-response study and n *in vivo* model. We analized the ability of *H. pylori* to colonize mice gastric mucosa following DHA, ST or a combination of both treatments. Our data demonstrate that DHA decreases *H. pylori* growth *in vitro* in a dose-dependent manner. Furthermore, DHA inhibits *H. pylori* gastric colonization *in vivo* as well as decreases mouse gastric mucosa inflammation. Addition of DHA to ST was also associated with lower *H. pylori* infection recurrence in the mouse model. In conclusion, DHA is an inhibitor of *H. pylori* growth and its ability to colonize mouse stomach. DHA treatment is also associated with a lower recurrence of *H. pylori* infection in combination with ST. These observations pave the way to consider DHA as an adjunct agent in *H. pylori* eradication treatment.

## Introduction

*Helicobacter pylori* infection is extremely common world-wide with more than two thirds of the world population infected. This gram-negative bacterium is recognized as a major etiological factor in chronic active gastritis, gastric duodenal ulcers and gastric cancer. Successful treatment of this pathogen often leads to regression of some of its associated diseases [1]. The outcome of the infection depends on the complex interaction established between the bacteria and its host, including the virulence of the infecting strain and the genetic factors and age of the host. Environmental factors, mainly associated to diet, also contribute to this complex interplay [2].

*H. pylori* eradication treatment has not changed to a large extent in the last decades. It relies on a triple therapy approach that combines clarithromycin or metronidazole in combination with other antibiotics and acid inhibitors [3]. However, this treatment regimen raises some concern mainly due to possible recurrence of infection, high cost, side effects, poor compliance to therapy and most importantly, acquired resistance to classically used antibiotics [4]–[8]. In fact, it has been estimated that eradication therapy is unsuccessful in approximately one in every five patients [4], [6], [8]–[9]. Therefore a proper regimen should have high efficacy against clarithromycin and metronidazole-resistant strains of *H. pylori* because these strains are increasingly encountered in routine clinical practice.

It has been proposed that certain polyunsaturated fatty acids (PUFA) hold an inhibitory effect on bacterial growth [10]–[15]. Some mechanisms have been reported for PUFAs bacteria inhibitory action and gastric protective effect. These include the ability to disrupt cell membrane leading to bacteria lysis [15], and the ability to modulate the synthesis of mucosal anti-inflammatory prostaglandins, such as Prostaglandin E_2_ (PGE2) [16]. The decline in duodenal ulcer incidence associated with the rise in dietary intake of PUFA, independently of *H. pylori* treatment, led to a growing interest in the role of these fatty acids [17]. Furthermore, it has been demonstrated that concentration of 2.5×10^−4^ M of Linoleic acid (n-6 PUFA) could inhibit the growth of *H. pylori in vitro*. This inhibitory effect is thought to be related to the extent of unsaturation within PUFA [10]. Despite the documented anti-microbial effect of PUFAs on the growth of fungi, protozoan, viruses and various types of bacteria [3], [18], few studies have described their action on *H. pylori* growth and viability.

To our knowledge, no studies have investigated the effects of docosahexaenoic acid (DHA), a highly unsaturated PUFA present in fish oil, on *H. pylori* growth *in vitro* and most importantly *in vivo* on its ability to colonize gastric mucosa. Our general aim was therefore to assess the effect of DHA on *H. pylori* growth using both *in vitro* and *in vivo* models. We performed an *in vitro* dose-response study of *H. pylori* growth inhibition by DHA, as well as an analysis of DHA effectiveness in inhibiting *H. pylori* gastric mucosal colonization in a mouse model. We also compared the effectiveness of a standard therapy (ST) combined with DHA in *H. pylori* eradication and recurrence success.

## Methods

### Fatty acids, *H. pylori* strains and culture conditions

DHA was obtained from *Cayman Chemical Company* (Michigan, USA) with a degree of purity of 99% in ethanol 0.06%. The *H. pylori* strains used were: SS1 [19], B128 [20] and 26695 (ATCC 700392) obtained from the American Type Culture Collection (ATCC, Rockville, MD). *H. pylori* was grown on blood agar base 2 (Oxoid, Lyon, France) plates supplemented with 10% defibrinated horse blood (bioMérieux, Marcy l'Etoile, France). Plates were incubated at 37°C for 24 to 48 h under microaerobic conditions (7% O_2_, 10% CO_2_; Campygen gas pack; Oxoid). To determine growth kinetics, plate-grown *H. pylori* strains were inoculated to an initial optical density at 600 nm (OD600) of 0.03 into liquid Brucella broth (BB) (Oxoid) supplemented with 10% fetal calf serum (FCS).

*H. pylori* grown for 18–20 hours yield a viable count of approximately 5.64×10^8^ colony forming units (CFU)/ml.

### DHA treatment of *H. pylori* cultures

Stock solutions of DHA were diluted in BB enriched with 10% FCS and used from 50 µM to 1000 µM. To establish *H. pylori* growth curves, 18–20 hours bacteria cultures were diluted 100-fold in 10 ml of medium with or without DHA to an initial OD of 0.03. Each experiment, consisting of a control (non-treated *H. pylori* culture) and *H. pylori* incubated with DHA at 50 µM, 100 µM, 250 µM, 500 µM and 1000 µM was performed in triplicate. *H. pylori* broth cultures were incubated under microaerophilic conditions, as described above. Every 6 hours, during a 48 hours period, a 200 µL sample of each bacterial culture was isolated, the OD measured and aliquots serially diluted and plated on blood agar petri dishes. After 48 hours of incubation, the number of viable bacteria was determined by colonies forming unit (CFU) counting.

The ID50 values were determined for the three strains every 12 hours until 72 hours of growth in the presence of increasing concentrations of DHA from 50 to 1000 µM. ID50 corresponds to the DHA dose that leads to 50% of bacteria survival characterized by the ratio between the number of viable bacteria at a certain dose of DHA compared to the number of total bacteria in the control culture at the same time-point.

### Electron microscopy analysis

In order to examine the effect of DHA treatment on *H. pylori* structure and morphology, strains 26695 and SS1 were grown for 12 hours in the presence of DHA 100 µM, as described. Then, control and DHA treated *H. pylori* cultures were observed by scanning electron microscopy. Briefly, samples were washed in phosphate buffered saline (PBS) and fixed in 2.5% glutaraldehyde for 30 min. Fixed bacteria were dehydrated in ethanol and treated with hydroxymethyl disilazane dried over a glass coverslip and sputter-coated with gold-palladium. The resulting samples were examined with a FEG-SEM JEOL 7001F electron microscope.

### Mouse infection and DHA treatments

Mouse infection studies were carried out in strict accordance with the recommendations in the Specific Guide for the Care and the Use of Laboratory Animals of the *Institut Pasteur*, according to the European Directive (2010/63/UE) and the corresponding French law on animal experimentation (Arrêtés de 1988). The protocol used in the present study was approved by the Committee of Central Animal Facility Board.

Six-week-old specific pathogen-free (*H. pylori* free) C57BL/6 male mice (Charles Rivers, France) were orogastrically inoculated with 100 µL of a suspension of 10^8^ CFU/mL of *H. pylori* strain SS1 known to colonize efficiently the mouse gastric mucosa, whereas control groups of mice were given peptone trypsin broth alone. This is a well-established mouse model of *H. pylori* infection [21]–[22]. The experiment consisted of four groups of twenty-four mice. Each group was further divided into four groups of six mice: control group (given peptone trypsin broth alone), non-infected DHA-treated group, *H. pylori* infected group and *H. pylori* infected and DHA-treated group. Mice from DHA treated groups received drinking water supplemented with 50 µM of DHA, 24 hours after *H. pylori* SS1 infection and throughout the entire experiment. The DHA concentration used was determined according to the notion that the maximum daily recommendation for total n-3 PUFA in humans, is between 1–2 g/day (50 to 100 µM in the gastric milieu). To prevent any degradation of DHA due to light, the supplemented drinking water was contained in dark-coloured bottles. Toxicity signs associated to DHA treatment in mice were checked during the experiment (weight variation, appetite and liver-gastric histopathology). No toxic effects were observed: no weight variations (Figure S1) and no signs of liver toxicity as indicated by histopathology analysis (data not shown). At each time-point (one, three, six and nine months) six mice from each group were sacrificed, stomachs isolated and *H. pylori* colonisation measured as previously described [22]. Since *H. pylori* is a human-specific bacteria that is not able to colonize the mouse stomach unless inoculated, no animals were sacrificed at the time-point zero. The number of viable bacterial colonies was counted and expressed as CFU per gram of gastric tissue.

### Mouse DHA and Antibiotics standard therapy treatment (ST)

The efficacy to eradicate *H. pylori* infection in mouse gastric mucosa was compared between a 7-days ST associated or not with a 15-days treatment of DHA 100 µM. This concentration of DHA was used in order to allow as much as possible DHA saturation in the mouse gastric lumen. No toxic side effects were observed in this condition of DHA treatment. The efficacy in preventing *H. pylori* recurrence following ST and/or DHA treatment regimen was also assessed prospectively (time-point 14 weeks). Twenty-four mice were infected by *H. pylori* strain SS1 for 4 weeks and divided into four groups of six animals. An additional group of non-infected and non-treated animals (n = 4) was given peptone trypsin broth alone as a control. Briefly, infected mice were dosed *p.o.* with omeprazole (400 µmol/kg/d; Sigma-Aldrich), metronidazole (14.2 mg/kg/d; Sigma-Aldrich), and clarithromycin (7.15 mg/kg/d) in a 0.1-ml volume daily for 7 days [23]. This antimicrobial regimen previously showed 100% eradication of *H. pylori* in C57BL/6 infected mice and it is referred to as ST [23]. Three therapeutic options were analyzed: ST *p.o.* for 7 days, DHA addition to drinking day water for 15 days, or a combination of both ST for 7 days, and at the same time DHA for 15 days. All treatments were administered at 4 weeks post-*H. pylori* infection. Mice were euthanized at 6 and 14 weeks post- infection; stomachs were isolated and *H. pylori* colonization was quantified as previously described [22].

### Mouse gastric histology

After 6 and 9 months of infection, mice stomachs were subjected to histology analysis. Biopsy specimens were fixed in 10% formalin routinely processed in paraffin. Four-µm sections were stained with hematoxylin/eosin. Gastric histology analysis was assessed for both *antrum* and *corpus* parts. The intensity of the lesions was semi-quantitatively classified according to *Eaton et al* [24]. Briefly, infiltrates of polymorphonuclear cells (PMN) and plasmocytes were graded as follows: 0 – no infiltrates; 1 – mild, multifocal infiltration; 2 – mild, widespread infiltration; 3 – mild, widespread and moderate multifocal infiltration; 4 – moderate widespread infiltration; 5 – moderate, widespread and severe multifocal infiltrations. Lymphoid (Linf) aggregates were graded 1 (mild, 1–10 glands), 2 (moderate, 10 to 20 glands) or 3 (severe, more than 20 glands).

### Measurement of prostaglandin-E2 (PGE2) in mouse serum

At each time-point of sacrifice (one, three, six and nine months) mice serum was collected and kept at −20°C, until further analysis. PGE2 levels were determined by enzyme immunoassay using a Prostaglandin-E2-Monoclonal Enzyme immunoassay Kit (R&D systems, Minneapolis, USA) according to the manufacturer's protocol.

### Statistical Analysis

Statistical analysis were performed with the R software [25] Data on the effects of DHA on *H. pylori* growth *in vitro* were analysed with a linear model using the “lm" function and default settings. For each strain the linear model included only the dose as qualitative variable. Then pairwise *t*-tests were performed to assess the significance of differences between doses, with a Hochberg p-value adjustment for multiple testing [26]

Results in mice experiments are expressed by median values as the data do not follow a normal distribution. Pairwise comparison Mann-Whitney U tests were used to assess differences in mouse gastric colonization by *H. pylori* when supplemented with DHA with a Hochberg *P*-value adjustment for multiple testing [26]. Kruskal-Wallis test was used to assess decreasing *H. pylori* recurrence rate with DHA addition to ST. All statistical tests were two-sided. Differences were considered significant for *P*<0.05. To assess the significance of anti-inflammatory effect of DHA in the infected gastric mucosa of mice, a student *t*-test was performed to compare control condition *vs* treated condition for each combination of time and stomach region considered. The four resulting p-values were then adjusted with the Hochberg method [26]. To analyze the differences in PGE2 production in infected mice treated with DHA compared to untreated, a linear model was fitted to the data including time and treatment as qualitative variables, as well as the interaction term.

## Results

### *H. pylori* growth is inhibited by DHA *in vitro*

**Figure 1:**
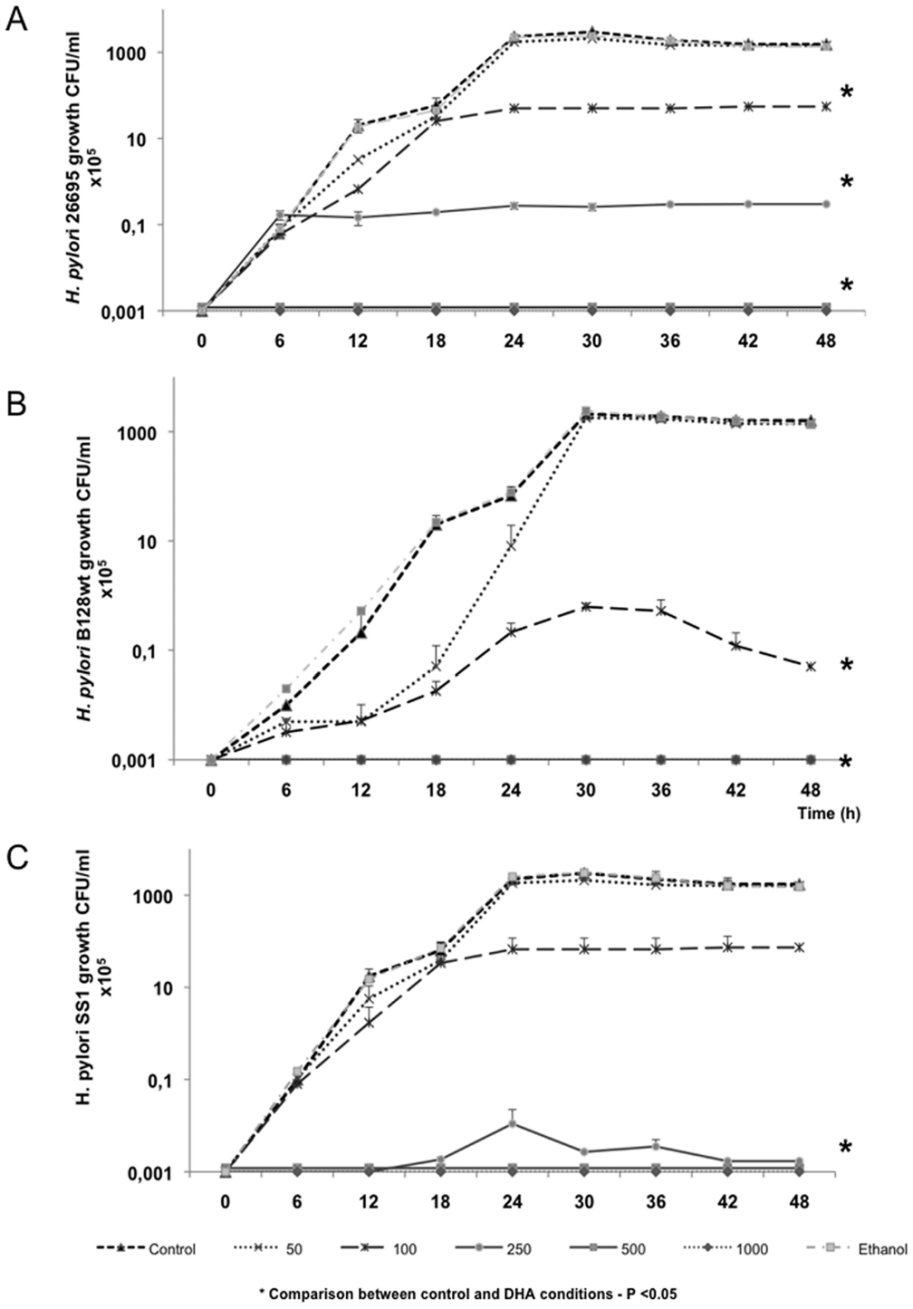
DHA effect on *H. pylori* growth. Growth of *H. pylori* strains A) 26695, B) SS1 and C) B128 during 48 hours in the presence of increasing concentrations of DHA from 50 to 1000 µM. Ethanol 0.06% v/v was used as a vehicle in DHA original stock solution, and therefore the presence of ethanol at the same concentration was also analyzed on *H. pylori* control culture with no effect on bacterial growth for the three strains. Data are expressed as the mean ± Standard Deviation and are representative of three independent experiments. * Refers to significant differences in *H. pylori* growth between controls and DHA-treated conditions (50 µM to 1000 µM of DHA).

In order to analyse DHA effects on *H. pylori* growth, three different bacterial strains 26695, SS1 and B128 were studied (Figure 1). *H. pylori* growth in controls was exponential until approximately 18–20 hours, then reaching a stationary phase. In the presence of DHA, the growth of these three strains was reduced with a statistically significant effect from DHA 100 µM (*P* = 2.86×10^−11^; *P* = 1.37×10^−08^; *P* = 9.64×10^−12^ for strains 26695, SS1 and B128 respectively). In addition, the statistical analysis of DHA effect between the different doses by pair-wise *t*-test showed a significant difference between bacterial growth observed at DHA 50 µM compared to the highest inhibitory doses of DHA from 100 µM to 1000 µM (4×10^−7^<*P*<6.4×10^−6^). Generation time during exponential growth phase (0–18 h) was longer for 100 µM of DHA in all strains compared to controls (*P*<0.05). At higher concentrations of DHA (250 µM or more) the effect on bacterial cultures was drastic with no growth observed for all strains, compared to control (*P* = 9.41×10^−12^; *P* = 1.36×10^−08^; *P* = 2.30×10^−12^ at DHA 250 µM, for strains 26695, SS1 and B128 respectively) (Figure 1).

The concentration required to inhibit growth of bacteria by 50% (ID50) at 48 hours of culture was 75 µM for both *H. pylori* strains 26695 and B128, and 85 µM for strain SS1 (Figure S1). The efficiency of DHA to inhibit *H. pylori* growth *in vitro* was therefore similar for all strains.

**Figure 2:**
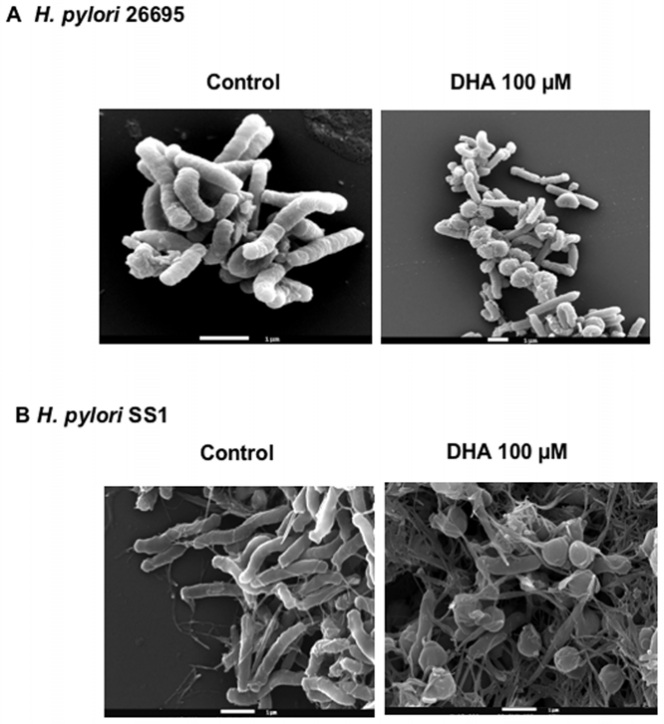
Alteration of *H. pylori* morphology upon DHA treatment. *H. pylori* strains A) 26695 and B) SS1 cultured for 12 hours incubated with 100 µM of DHA show changes in cell shape as observed by scanning electron microscopy. The presence of 100 µM of DHA leads to spherical/coccoids bacterial forms for both strains.

Scanning electron microscopy was used to analyse the morphology of *H. pylori* strains 26695 and SS1 following DHA treatment. After being treated with 100 µM of DHA, strains 26695 (figure 2A) and SS1 (figure 2B) showed a different morphology when compared to controls, as characterized by the presence of spherical/coccoid forms. Treatment with 250 µM DHA had a dramatic effect on the morphology of the few moribund bacteria present in the culture (data not shown). Thus, DHA is an efficient inhibitor of *H. pylori* growth *in vitro*, promoting the coccoid morphology of bacteria that is known to be a non-cultivable form [27].

### DHA inhibits *H. pylori* gastric colonization in mice

**Figure 3:**
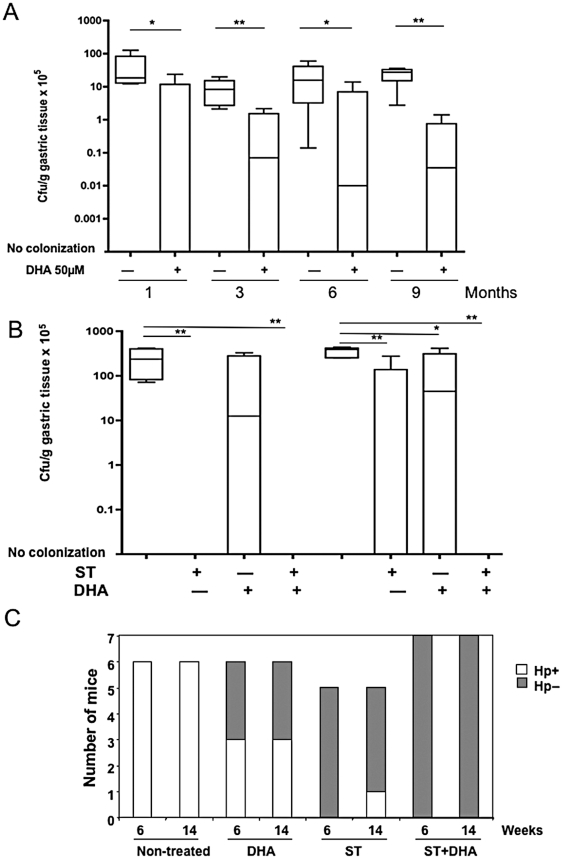
Inhibition of *H. pylori* SS1 gastric colonization by DHA treatment and a combination of standard antibiotherapy (ST) and DHA in C57BL/6 mice. **A**) Analysis of *H. pylori* SS1 gastric colonization inhibition by DHA treatment in C57BL/6 mouse over time. Four groups each encompassing six mice: one group of non-treated, non-infected controls; one group of animals infected by *H. pylori* strain SS1; one group of mice supplemented with 50 µM of DHA in the drinking water; and one group infected by *H. pylori* strain SS1 and supplemented with 50 µM of DHA in the drinking water as described in materials and methods. The non-infected control group and DHA treated mice are not colonized and are not reported in the figure. In each condition mice were sacrificed after one, three, six and nine months. Median values of gastric colonization between *H. pylori* infected mice non-DHA treated and DHA treated were 18.36×10^5^ *vs* 0.0049×10^5^, 8.24×10^5^ *vs* 0.069×10^5^, 15.71×10^5^ *vs* 0.0096×10^5^ and 27.45×10^5^ *vs* 0.034×10^5^ cfu/g of gastric tissue after 1, 3, 6 and 9 months, respectively. * P<0.05; **P<0.005. **B**) Analysis of the effect of the Standard antibiotics Therapy (ST) on the level of gastric mucosa colonization by *H. pylori* infection compared with DHA treatment and DHA in combination with ST in mice. Four groups of mice, each encompassing six (n = 6) were infected by *H. pylori* strain SS1. Control group (n = 4) was given peptone trypsin broth alone. As described in materials and methods, three therapeutic options were given to mice: ST *p.o.* for 7 days, DHA addition to mice drinking day water for 15 days, or a combination of both ST for 7 days, and at the same time DHA for 15 days. All treatments were administered at 4 weeks post *H. pylori* infection and gastric colonization measured at 6 and 14 weeks post infection. * P<0.05; **P<0.01. **C**) Number of mice colonized and not colonized by *H. pylori* under the different conditions of treatment described above. Open bars represent the number of mice infected by *H. pylori* (Hp^+^), whereas grey bars represent the number of non-infected mice (Hp^−^). The Kruskal-Wallis test was used to assess the consequences on *H. pylori* mouse gastric colonization of DHA treatment. Our results show a significant (*P* = 0.0036) reduced rate of recurrence of *H. pylori* infection in animals supplemented with DHA.

In order to assess the effectiveness of DHA as a therapeutic agent, we investigated the effect of exposure to DHA on the *H. pylori* colonization of mouse gastric mucosa. The ability of *H. pylori* strain SS1 to colonize the gastric mucosa was significantly decreased in 50 µM DHA-treated mice as compared to mice from the infected non-treated group at each time-point of infection from 1 to 9 months (*P* values: 0.028; 0.023; 0.024 and 0.020 at 1, 3, 6 and 9 months respectively) (Figure 3A). In the non-treated group, all animals were successfully colonized by *H. pylori*. In contrast, amongst DHA supplemented-mice, only 50% of animals are infected, with a median value for the gastric colonization lower than 3 to 4 Log compared to the infected and non DHA-treated group at the same time-point. No toxic effects were observed due to DHA treatment: no weight variations (Figure S2) and no signs of liver toxicity as indicated by histopathology analysis (data not shown). These data showed that also *in vivo*, DHA inhibits efficiently *H. pylori* gastric colonization.

### DHA and ST treatments

It has already been shown that triple therapy consisting of omeprazole plus two antibiotics (clarithromycin and metronidazole) is 100% efficient in eradicating *H. pylori* in the mouse gastric mucosa [23]. We confirmed ST 100% efficacy in our mouse model: no colonization in the group of mice treated with ST at the 6 weeks time-point (*P* = 0.01) which corresponds to one week after the end of ST treatment (Figure 3B). It is known that subsequently to the antibiotic treatment against *H. pylori* infection there is a significant percentage of relapses. Therefore, our aim was to compare prospectively the rate of *H. pylori* infection recurrence between ST and ST+DHA treatment regimens. Mouse gastric colonization was compared after ST, DHA or ST+DHA treatments at the time-points 6 and 14-weeks that correspond to 4 weeks of infection followed by 2 and 10 weeks after the end of treatment, respectively. At the time-point 6 weeks, although not reaching statistical significance, a decrease in the gastric colonization is observed in DHA treated mice. As previously mentioned ST was 100% efficient in inhibiting colonization (Figure 3B), with none of the infected mice presenting *H. pylori* viable colonies in the gastric mucosa. At 14-weeks the presence of DHA had a significant inhibitory effect on the *H. pylori* infection recurrence, whether used alone (*P* = 0.04) or as an adjuvant to ST (*P* = 0.0028), as compared to infected mice untreated (Figure 3B). Among the six mice receiving ST+DHA at 14 weeks post infection, none showed viable *H. pylori* in their gastric mucosa, compared to 1 among 5 (20%) and 3 among 6 (50%) (Median level: 4.5×10^6^ CFU/g; SD 4.1) mice in the group treated with ST and DHA alone, respectively (Figure 3C). At this time-point the median value of gastric colonization level in non-treated *H. pylori* infected mice was 1.7×10^6^ CFU/g. These data showed that a combination of ST plus DHA is very efficient to prevent *H. pylori* infection recurrence.

### Mouse gastric mucosa inflammation status

**Figure 4:**
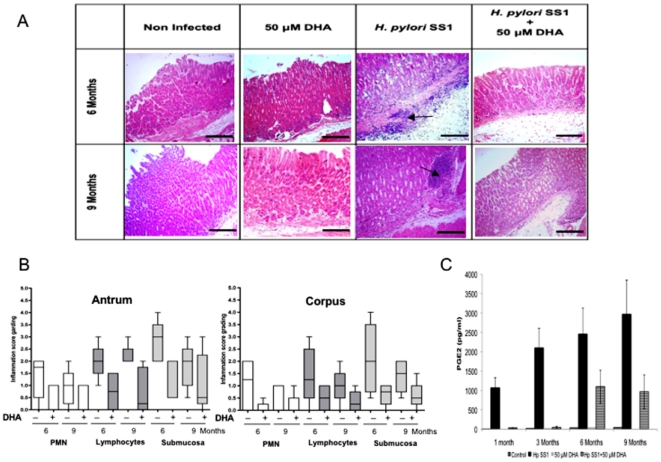
Anti-inflammatory effects of DHA in the infected gastric mucosa of mice. **A**) Histological analysis of inflammatory lesions of the gastric mucosa of *H. pylori* infected or non-infected mice treated or not with DHA 50 µM after 6 and 9 months. No differences were observed between non-infected mice DHA-treated or non-treated. In infected mice, the DHA treatment in infected mice leads to a decrease of the gastric mucosa thickness compared to *H. pylori* infected mice but non-DHA treated at both time-points. Infiltrates of polymorphonuclear (PMN) cells and plasmocytes as well as number of lymphoid aggregates (arrows) were lower in *H. pylori* infected mice treated with DHA compared to infected mice non-treated. The infection leads to the formation of lymphoid aggregate (arrows) not observed in mice treated with DHA. Scale bars correspond to 100 µm. **B**) Semi-quantification of inflammation score grading in the gastric mucosa. At each time-point, 6 and 9 months, mean score grading for each group of mice are higher in the *antrum* than *fundus* part. The presence of DHA leads to a significant decrease of the *antrum* inflammation of the *H. pylori* infected gastric mucosa. PMN: Polymorphonuclear cells; Linf: Lymphocytes aggregates; Submuc: Submucosa. Comparison was made between infected mice non-DHA supplemented and infected mice DHA supplemented. C) Measurement of PGE2 in the mice serum. Lower PGE2 values were observed in infected mice that received DHA 50 µM compared to infected mice non-DHA treated (adjusted *P* values 0.016 at 1 month; 0.008 at 3 months; 0.054 at 6 months; 0.012 at 9 months). PGE2 levels were lower at 6 months in DHA-treated mice compared to untreated mice, even though the difference cannot be considered statistically significant. Control group corresponds to non-infected and non-DHA treated mice. Bars represent means with standard deviation.

Mouse gastric mucosa at 1 and 3 months of *H. pylori* infection, did not present significant signs of inflammation. However, at 6 and 9 months post-infection, the gastric mucosa of infected-mice present important inflammatory lesions with infiltrates of polymorphonuclear cells (PMN) and plasmocytes as well as the presence of important lymphocytes aggregates (Figure 4A). Gastric inflammation and histology analysis revealed a significant lower inflammation in the gastric mucosa of H. pylori infected mice treated with DHA, compared to H. pylori infected mice non-treated after both 6 (*P* = 1.83×10^−6^) and 9 months (*P* = 0.0004) of infection (Figure 4B). In addition, according to the semi-quantitative evaluation of inflammatory lesions [24], infiltrates of PMN, plasmocytes and lymphoid aggregates were less abundant in DHA-treated mice than in non-DHA-treated ones (*P*<0.05) (Figure 4B). These differences were significant at 6 and 9 months in both parts of the stomach, the antrum (*P* = 1.47×10^−4^ and *P* = 1.4×10^−3^) and the corpus (*P* = 1.34×10^−3^ and *P* = 0.0485), respectively.

PGE2 is produced in several pathological conditions by a wide variety of tissues. Increased amount of PGE2 is associated with inflammation and tissue injury [28]. At both time-points, infected mice treated with DHA showed lower serum PGE2 levels compared with infected but non DHA-treated mice (*P* = 0.0284) (Figure 4C). Altogether, these data showed that in the mouse model of chronic *H. pylori* infection, DHA addition is accompanied by a reduction in the inflammatory response.

## Discussion

The present study identifies DHA as a novel antibacterial agent, which inhibits *H. pylori* growth *in vitro* and its ability to colonize the gastric mucosa in a mouse model. It was previously demonstrated that some free fatty acids can inhibit up to 50% of *H. pylori* growth *in vitro* within a concentration of 1 mM [13], whereas 2 mM would kill all bacteria [13], [15]. To our knowledge this is the first study demonstrating the inhibitory effect of DHA on *H. pylori* growth, both *in vitro* and most importantly *in vivo*. As we showed, DHA is able to inhibit *H. pylori* gastric colonization in a significant proportion of mice (50%, P<0.05). The lack of effect in all DHA-treated mice may be due to variation in the delivery of DHA to animals. However, biological variability within mouse population cannot be excluded. According to these data, DHA treatment should not be regarded as a replacement strategy for conventional antibiotic treatment. However, it is very interesting that in an experiment designed to evaluate DHA effectiveness as a co-adjuvant to ST antibiotherapy to prevent *H. pylori* infection recurrence, the addition of DHA yielded better results than ST alone. Thus, our results support the use of DHA in combination with standard antibiotic therapy for *H. pylori* treatment to inhibit efficiently the infection recurrence. These data suggests DHA supplementation as a safe prophylactic/preventive strategy for *H. pylori* infection eradication that is worth further consideration.

DHA concentration was a critical point in our study, as the concentration observed to be effective varied according to the experimental model used. In *in vitro* studies, differences in the level of sensitivity to DHA are observed between the three *H. pylori* strains tested. This may be due to the high genetic variability which characterizes *H. pylori* strains. Despite that, 100 µM of DHA is enough to significantly inhibit bacteria growth, with a maximum inhibitory effect at 250 µM. Hence, DHA concentrations have to be optimized for different experimental settings in order to obtain optimum effects. This is not surprising and it is actually typical of experiments aiming at measuring the effect of different drugs on microbiological agents [29]. *In vivo*, several parameters such as the biodistribution of the compounds, their chemical stability, their metabolization and their resistance to the extreme acidic conditions of the gastric lumen can modulate their bioactivity. Our *in vivo* analysis showed that the presence of DHA at a concentration of 50 µM in mice drinking water is efficient to inhibit significantly *H. pylori* gastric colonization. This concentration is within the range of concentrations reachable with diet in the human stomach for any PUFA [15]. Levels of DHA given to mice in our experiments are also within a safe and well tolerated range of concentrations with no toxic effects observed, in agreement with several clinical trials that have used DHA supplementation at higher doses with no reports of toxicological effects [30]–[31].

Despite the experimental data supporting an inhibitory effect of DHA on *H. pylori* growth, no association between DHA intake and *H. pylori* infection incidence has been made. In fact, it could be regarded as puzzling that countries like Japan and Portugal, with dietary habits including high intakes of fat fish naturally rich in DHA, exhibit some of the highest incidences of *H. pylori* infection worldwide. There are several explanations for this apparent discrepancy: 1- DHA daily intake recommendations in grams do not have a direct translation in terms of DHA gastric concentration. It is therefore not guaranteed that a DHA concentration of 50 µM will be achieved by food consumption, even when daily intake recommendations are met; 2- DHA is typically present in food as triacylglycerol that will be hydrolysed along the digestive tract, leading to only a small amount being released as free fatty acid in the gastric milieu [32]; 3- the high degree of unsaturation of the DHA molecule makes it vulnerable to oxidation and temperature. This can result in the modification of the DHA chemical structure, and interfere with its anti-*Helicobacter* properties. Hence, the level of fish consumption, or any other DHA-rich food for that matter, does not constitute an adequate measure of DHA gastric concentration and DHA availability to *H. pylori*. This point is well illustrated by the fact that although no association between fish intake and gastric cancer risk could be drawn from the Japanese population, the percentage of DHA in the membrane composition of erythrocytes was directly associated with a lower risk of developing gastric cancer [33]. These arguments may justify the lack of a positive association between *H. pylori* gastric colonization/gastritis and high levels of PUFA supplementation in clinical trials. As an example is a study in which *H. pylori* infected-patients were supplemented with capsules containing linolenic acid (n-3 PUFA), linoleic acid (n-6 PUFA) or subjected to an increased margarine intake in the diet [30]. Although PUFA overall levels were significantly increased in individuals supplemented with PUFAs in comparison with the control group, no *H. pylori* growth inhibition or lower inflammation status were observed.

The mechanism underlying the inhibitory effect of lipids on *H. pylori* growth is unknown. However, it has been shown that fatty acids, in concentrations ranging from 1 to 10 mM, affect the lipid constitution of the bacterial plasma membrane [18]. A possible explanation for the bactericidal effect of PUFA relies on the formation of toxic lipid peroxides generated from an oxidative process involving H_2_O_2_ and iron [10], [12]. Another potential mechanism suggested for the killing of bacteria after exposure to fatty acids is by cell membrane alteration, leaving the bacterial cell wall intact. According to this mechanism, fatty acids would be incorporated into the cell membrane resulting in an increased membrane fluidity and permeability; this effect would be further exacerbated by the presence of unsaturated double bounds, as they seem to exert a toxic effect towards the bacterial cell membrane [10]–[15]. Supporting this cell membrane disruption hypothesis, it has been demonstrated that radiolabeled PUFAs added to *H. pylori* liquid cultures are incorporated into the *H. pylori* plasma membrane [15]. In agreement, our electron microscopy observations revealed *H. pylori* morphological changes upon n-3 PUFA DHA (C22:6) treatment. A proportion of *H. pylori* -DHA treated bacteria displayed coccoid forms, known to be associated with a bacterial decreased viability [27]. The morphological transition of *H. pylori* from a bacillary to a coccoid shape has been previously reported to be related to modification of the bacterial cell wall peptidoglycan [34].

In summary, our results show that DHA inhibits *H. pylori* growth both *in vitro* and *in vivo*. Although the mechanisms underlying this inhibitory effect are still unclear, our results demonstrate that DHA impairs *H. pylori* growth and gastric colonization and attenuates the host inflammatory response. More importantly, the combination of DHA and ST strongly decreased the recurrence of *H. pylori* infection in the mouse model. Although not evaluated in this study, the effectiveness of ST/DHA combined treatment in inhibiting *H. pylori*-induced gastric inflammation is another important aspect of the benefit of DHA therapy that deserves to be further addressed. It should be emphasized that DHA raises low toxicological constraints and it is also well tolerated. In agreement, it is widely and commonly used both at a clinical trial and at a diet level for long time periods. Additionally, DHA has cytoprotective properties and enhances the formation of prostaglandins [19].

Although the established therapeutical regimens for *H. pylori* eradication have been proven effective, treatment failure still occurs and is increasingly common [3]. Altogether the data obtained in the current work may pave the way to propose the use of DHA in preventive and curative strategies for *H. pylori* eradication, as a co-adjuvant agent in combination with standard therapy. Consequently, DHA supplementation within the range of concentrations we have tested should be considered as a safe prophylactic/preventive strategy against *H. pylori* infection.

## Supporting Information

Figure S1 **Growth of** ***H. pylori*** **strains 26695, SS1 and B128 during 72 hours in the presence of increasing concentrations of DHA from 50 to 1000 µM.** Data are expressed as *H. pylori* viability upon DHA treatment reported for bacterial cultures sampled every 12 hours. The number of total viable bacteria in the control cultures corresponded to 100% survival.
(DOCX)

Figure S2 **Mice weight variation during** ***H. pylori*** **infection and DHA treatment.** Four groups each encompassing six mice: one group of non-treated, non-infected controls; one group of animals infected by *H. pylori* strain SS1; one group of mice supplemented with 50 µM of DHA in the drinking water; and one group infected by *H. pylori* strain SS1 and supplemented with 50 µM of DHA in the drinking water as described in materials and methods. Data from the control group and DHA treated mice are not colonized as expected and not reported in the figure. These four groups of animals were used during the course of our experiment at every time-point: one, three, six and nine months. Regardless infection status and DHA treatment, all mice were weighted at each time-points of sacrifice. Results are represented as percentage of control group values. *t*-test analysis showed no significant differences over time amongst mice weight with DHA treatment and with or without *H. pylori* infection compared to controls. Bars represent standard deviation.
(DOCX)
