# Supplementary material for: Docosahexaenoic Acid Inhibits Helicobacter pylori Growth In Vitro and Mice Gastric Mucosa Colonization
Source: PLoS One. 2012 Apr 17;7(4):e35072. doi: 10.1371/journal.pone.0035072 (PMC3328494; doi:10.1371/journal.pone.0035072)

**Supplementary Figure legend Figure S1 –** Growth of *H. pylori* strains 26695, SS1 and B128 during 72 hours in the presence of increasing concentrations of DHA from 50 to 1000 µM. Data are expressed as *H. pylori* viability upon DHA treatment. Growth of control conditions were used as reference (100%).


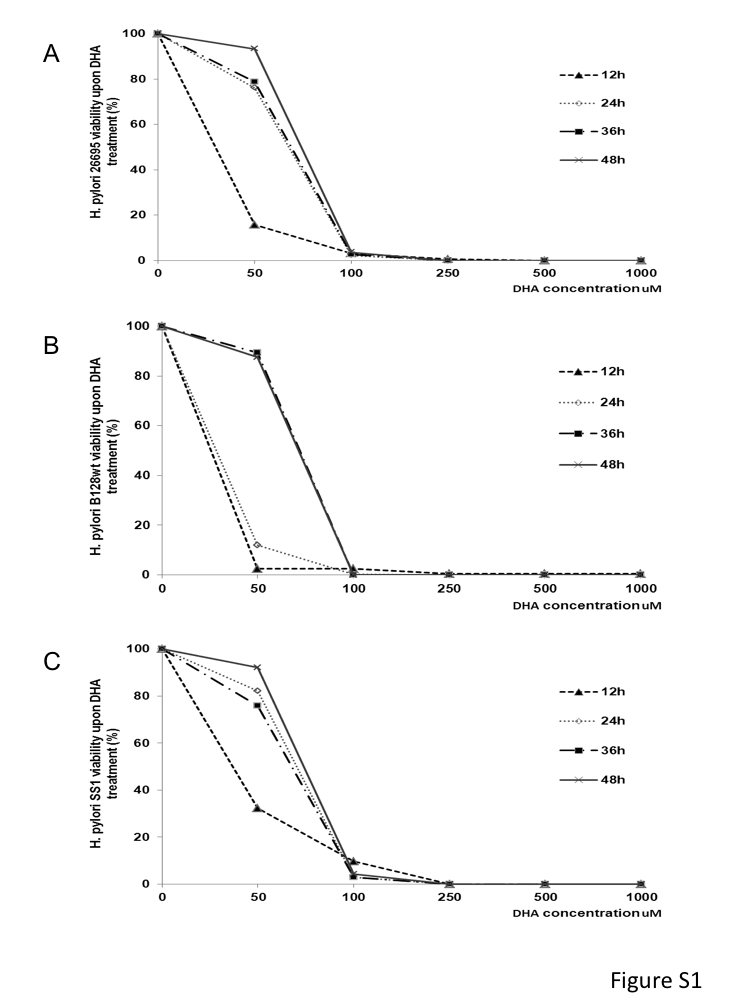

Supplement: Figure S1 — Growth of H. pylori strains 26695, SS1 and B128 during 72 hours in the presence of increasing concentrations of DHA from 50 to 1000 µM. Data are expressed as H. pylori viability upon DHA treatment reported for bacterial cultures sampled every 12 hours. The number of total viable bacteria in the control cultures corresponded to 100% survival. (DOCX) [file pone.0035072.s001.docx]
