# Supplementary material for: Docosahexaenoic Acid Inhibits Helicobacter pylori Growth In Vitro and Mice Gastric Mucosa Colonization
Source: PLoS One. 2012 Apr 17;7(4):e35072. doi: 10.1371/journal.pone.0035072 (PMC3328494; doi:10.1371/journal.pone.0035072)

**Figure 2S - Mice weight variation during *H. pylori* infection and DHA treatment.** Four groups each encompassing six mice: one group of non-treated, non-infected controls; one group of animals infected by *H. pylori* strain SS1; one group of mice supplemented with 50 µM of DHA in the drinking water; and one group infected by *H. pylori* strain SS1 and supplemented with 50 µM of DHA in the drinking water as described in materials and methods. Data from the control group and DHA treated mice are not colonized as expected and not reported in the figure. These four groups of animals were used during the course of our experiment at every time point: one, three, six and nine months. Regardless infection status and DHA treatment, all mice were weighted at each time points of sacrifice. Results are represented as percentage of control group values. t-test analysis showed no significant differences over time amongst mice weight with DHA treatment and with or without *H. pylori* infection compared to controls. Bars represent standard deviation.


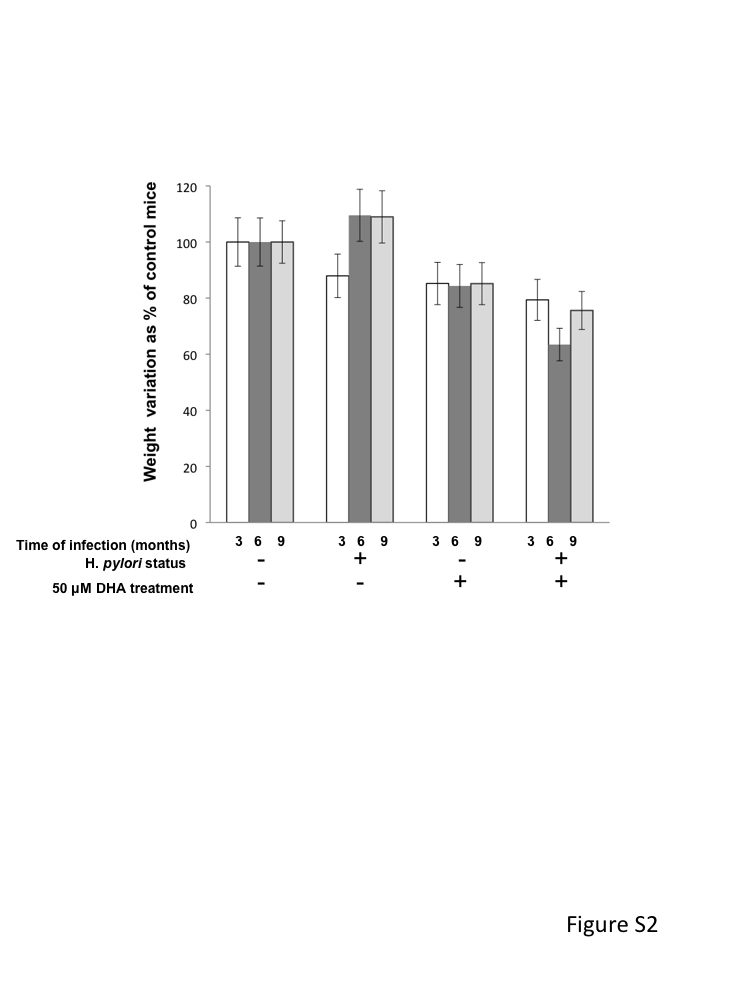

Supplement: Figure S2 — Mice weight variation during H. pylori infection and DHA treatment. Four groups each encompassing six mice: one group of non-treated, non-infected controls; one group of animals infected by H. pylori strain SS1; one group of mice supplemented with 50 µM of DHA in the drinking water; and one group infected by H. pylori strain SS1 and supplemented with 50 µM of DHA in the drinking water as described in materials and methods. Data from the control group and DHA treated mice are not colonized as expected and not reported in the figure. These four groups of animals were used during the course of our experiment at every time-point: one, three, six and nine months. Regardless infection status and DHA treatment, all mice were weighted at each time-points of sacrifice. Results are represented as percentage of control group values. t-test analysis showed no significant differences over time amongst mice weight with DHA treatment and with or without H. pylori infection compared to controls. Bars represent standard deviation. (DOCX) [file pone.0035072.s002.docx]
